# Novel, rapid, and reliable typing of vancomycin-resistant *Enterococcus faecium* CC17/ST80 strains using MALDI-TOF MS

**DOI:** 10.1128/spectrum.02702-25

**Published:** 2025-10-01

**Authors:** Silke Huber, Christina Brühwasser, David Eisele, Cornelia Lass-Flörl, Stefan Fuchs, Miriam Govrins

**Affiliations:** 1 Institute of Hygiene and Medical Microbiology, Medical University of Innsbruck 27280, Innsbruck, Austria; 2 Infection Prevention and Hospital Hygiene, University Hospital Innsbruck, Innsbruck, Austria; University of Guelph College of Biological Science, Guelph, Ontario, Canada

**Keywords:** vancomycin-resistant *Enterococcus faecium*, rapid bacterial typing, MALDI-TOF MS, clinical diagnostics, hospital hygiene

## Abstract

Vancomycin-resistant *Enterococcus faecium* (VREfm) is an important nosocomial pathogen. The recent emergence of the highly virulent clonal complex 17 (CC17) is posing a challenge for both therapeutic interventions and hospital infection control measures. Hence, prompt discrimination of CC17 VREfm from unrelated and less-virulent VREfm strains is essential for preventing its spread in hospitals and beyond. Between January 2022 and November 2024, 340 VREfm primary isolates have been identified in our lab and underwent genotyping by pulsed-field gel electrophoresis (PFGE) to survey a potential outbreak in the Tyrol region. In addition, whole-genome sequencing (WGS) was performed on a selected subset (*n* = 40). To curtail the lengthy time-to-result (TTR) of these methods, a novel typing protocol using matrix-assisted laser desorption/ionization time-of-flight mass spectrometry (MALDI-TOF MS) was established, validated, and optimized for rapid sample processing. PFGE and WGS showed that 61.2% of isolates (*n* = 208) belonged to a specific VREfm cluster identified as CC17 sequence type (ST) 80 *vanA* VREfm. A comprehensive MALDI-TOF MS analysis identified a distinct peak pattern specific to this lineage. This phenotypic characterization was used as a novel typing method with excellent performance (sensitivity: 1.00 [0.98−1.00], specificity: 0.89 [0.70−0.97]) and demonstrated a short TTR of 1 day after the cultural growth of VREfm. A rapid and novel MALDI-TOF MS-based typing approach for a specific CC17/ST80 *vanA* VREfm cluster was developed and enabled real-life application in routine diagnostics to assure accurate infection prevention and control measures. Future outbreak investigations may benefit from adopting this cost- and labor-efficient approach.

**IMPORTANCE:**

This study addresses the urgent need for faster ways to detect problematic hospital bacteria. A highly transmissible strain of *Enterococcus faecium* (CC17) has been spreading in healthcare settings, making infections harder to treat and control. Traditional methods to identify and track outbreaks are accurate but slow and resource-intensive, delaying critical infection control actions. By developing and validating a new method using matrix-assisted laser desorption/ionization time-of-flight mass spectrometry, the researchers demonstrated that this strain can be identified quickly, reliably, and at lower cost. Importantly, the new approach delivers results within a day, compared to the lengthy turnaround times of existing methods. This rapid detection tool provides hospitals with a practical solution to respond to outbreaks more effectively, prevent further spread, and protect vulnerable patients. The findings highlight a valuable step forward in strengthening hospital infection control and improving patient safety.

## INTRODUCTION

Vancomycin-resistant *Enterococcus faecium* (VREfm) commonly causes hospital-acquired infections with limited treatment options. Among different VREfm strains, the clonal complex 17 (CC17) family is a prominent lineage in hospital outbreaks and comprises several sequence types (STs), including the frequently detected ST80 and ST117 strains (1, 2). CC17 strains have been identified in various countries in and outside of hospital environments, with numerous genes and mobile elements mediating antimicrobial resistance as well as virulence factors enabling a more rapid spread and longer persistence compared to other *E. faecium* strains (1, 3, 4). Moreover, infections with VREfm CC17 are associated with high morbidity and mortality, especially in immunocompromised patients. This, combined with the high transmissibility in hospital environments, is particularly worrisome (5).

Bacterial genotyping is an indispensable tool in outbreak investigations, predominantly to identify routes of transmission. Pulsed-field gel electrophoresis (PFGE) used to be the “gold standard” for genotyping, but is now more and more replaced by whole-genome sequencing (WGS) approaches (6). WGS provides additional information about antimicrobial resistance and virulence and allows better reproducibility. Matrix-assisted laser desorption/ionization time-of-flight mass spectrometry (MALDI-TOF MS) has previously been used for bacterial typing, however for VREfm thus far with limited applicability (7, 8). A recent emergence of VREfm in the region of Tyrol, Austria, forced the implementation and validation of a real-life application of a novel and rapid MALDI-TOF MS-based phenotypical outbreak tracking tool. This cost-efficient method supported the prospective and accurate differentiation of outbreak and unrelated VREfm strains and hence may complement or replace conventional genetic typing methods.

## MATERIALS AND METHODS

### Clinical isolation, identification, and antimicrobial susceptibility testing (AST)

*E. faecium* was routinely isolated from clinical specimens. Species identification was performed using MALDI-TOF MS (Bruker Daltonik, Bremen, Germany) using the Biotyper library v.4.1 as reference. AST was conducted by disk diffusion following EUCAST guidelines (9) and breakpoints (10). All primary VREfm isolates were cryopreserved until further outbreak investigation.

### Strain typing by PFGE

Molecular typing was conducted using PFGE following the guidelines established by the Centers for Disease Control and Prevention (CDC) (11) and analyzed as previously described (12). Briefly, bacterial DNA was digested using the restriction enzyme SmaI (Promega GmbH, Walldorf, Germany) and strain-relatedness was assessed by visual analysis of PFGE restriction patterns, supplemented by computational band variation analysis using Bionumerics Applied Maths NV v.7.5 software (bioMérieux, Craponne, France).

### Strain typing by WGS analysis

WGS on a subset of VREfm isolates (*n* = 40) was performed by a commercial provider (ARES Genetics GmbH, Vienna, Austria). Multilocus sequence typing (MLST) profiles were determined in the primary analysis as well as in-house using PubMLST (13). For the latter, a total of 1,360 VREfm genomes were downloaded from NCBI and annotated using Seemann et al.’s MLST program (14) to determine outbreak strains and their distinct gene pattern. A single-nucleotide polymorphism (SNP)-based phylogeny for the forty VREfm isolates as well as 394 external VREfm genomes with the most similar profile was created using the program kSNP4 (15). Blast+ (16) was used to detect the presence/absence of various genes and plasmids to determine a presence/absence pattern unique to outbreak cluster, which could form the basis for the development of a strain-specific PCR (Table S1).

### Establishment and robustness of strain typing by MALDI-TOF MS

**Fig 1:**
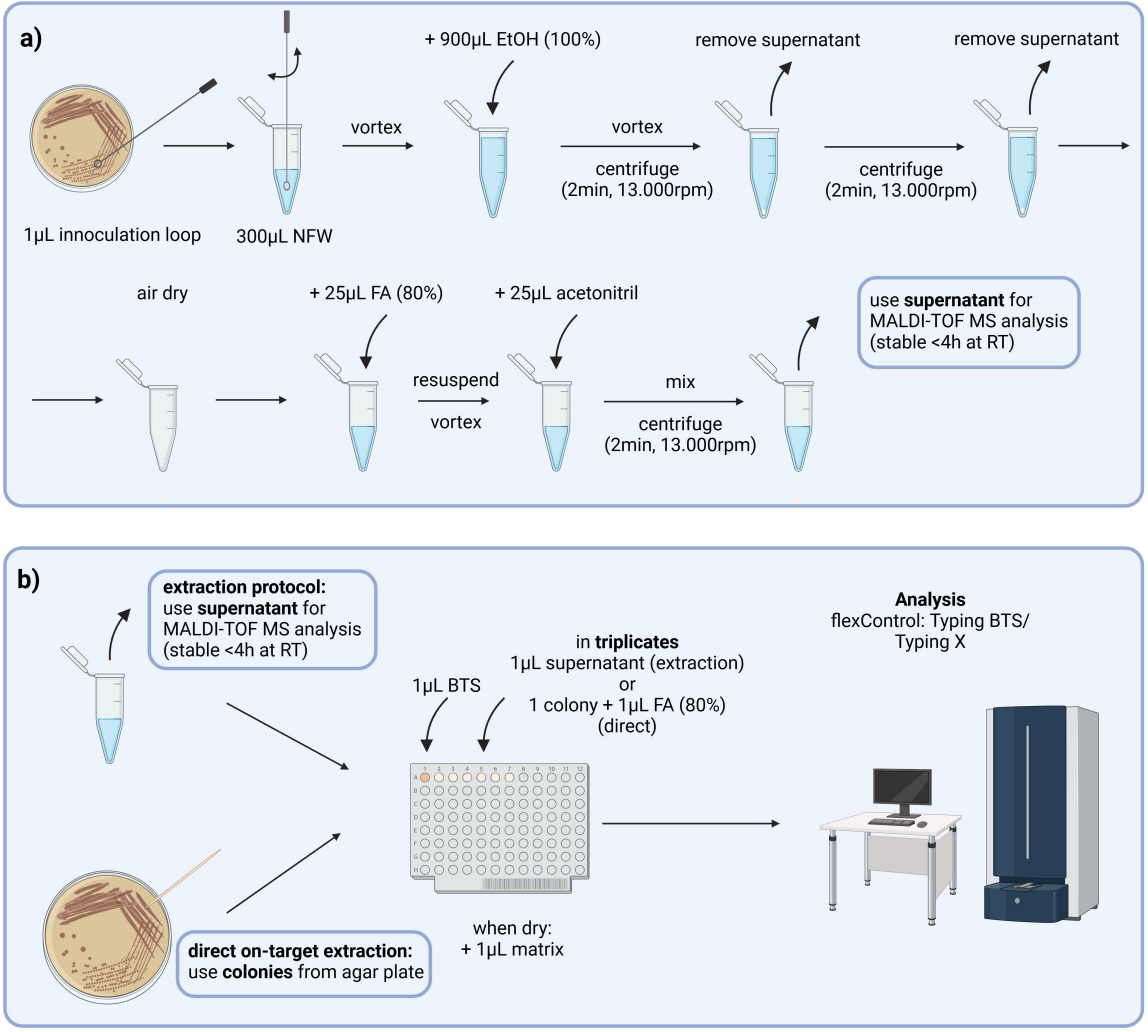
Rapid typing of vancomycin-resistant *Enterococcus faecium* (VREfm) by MALDI-TOF MS. (**a**) Sample preparation included an ethanol/formic acid protein extraction of VREfm isolates as recommended by the manufacturer (Bruker), before (**b**) extracts were prepared on targets and measured by MALDI-TOF MS (FlexControl v4.3). Mass spectra were analyzed by manually checking for the absence or presence of peaks discriminating isolates belonging to the outbreak cluster or independent isolates (FlexAnalysis v4.3). Later on, an optimized protocol was established using direct on-target protein extraction. BTS, bacterial test standard; FA, formic acid; NFW, nuclease-free water; RT, room temperature. Created in BioRender. Govrins, M. (2025). https://BioRender.com/c57w743.

A representative selection of previously analyzed VREfm isolates (56 clinical, 4 environmental) was used to establish MALDI-TOF MS-based typing method. Sample preparation included (i) the cultivation of cryopreserved VREfm isolates on selective agar (chromID VRE Agar, bioMérieux) (37°C, 48 h), (ii) a second cultivation step on Columbia III blood agar (BD) (37°C, 18 h), and (iii) an in-tube ethanol/formic acid extraction according to the manufacturer’s instructions (IFU MBT Compass HT IVD User Manual, Bruker) (Fig. 1). Extracts were transferred on a steel MALDI-TOF MS target plate (in triplicate) and each spot was covered with HCCA matrix (Bruker). Mass spectra of each sample were obtained in three independent measurements using the FlexControl software v4.3 (MBT_AutoX_Typing_by_ToM.axe, Bruker).

Data analysis was performed using FlexAnalysis v4.3 (Bruker). First, “baseline subtraction” and “smooth” functions were applied to align spectra baselines and filter background noise, respectively. Second, an internal calibration was administered to align all mass spectra to one common VREfm-specific peak at 6,342 *m/z*. In order to find a peak pattern specific to the outbreak strain, an initial visual inspection was focused on (i) identifying reliable peaks within repeated measurements and (ii) on discriminatory peaks between CC17/ST80 outbreak strains and unrelated strains. After identification of these specific peaks, a Python code was scripted to automatically screen the exported mass lists for these peaks using the Spyder software v6.0 (17). Further evaluation of the method was performed using a larger sample set (*n* = 278).

### Optimization and real-life application of VREfm strain typing by MALDI-TOF MS

Additionally, a direct on-target protein extraction with 1 µL formic acid (80%) was performed to shorten the sample preparation prior to MALDI-TOF MS analysis (*n* = 72) (Fig. 1b). Sensitivity, specificity, and accuracy of the optimized workflow compared to PFGE were calculated. Furthermore, an alternative agar plate (Müller-Hinton II agar, Becton Dickinson GmbH, Vienna, Austria) for bacterial cultivation was tested for its effect on the MALDI-TOF MS results (*n* = 20).

### Statistical analysis

Descriptive statistics are presented as mean ± standard deviation (SD). The diagnostic performance of the MALDI-TOF MS typing method was assessed by calculating sensitivity, specificity, and accuracy compared to PFGE as the gold standard. 95% confidence intervals (CIs) were calculated using the Wilson Score or the Clopper-Pearson method as appropriate. Statistical analyses were conducted with IBM SPSS Statistics v29.0 (IBM, NY, USA).

## RESULTS

### VREfm epidemiology

From January 2022 to November 2024, a total of 2,157 *E. faecium* primary isolates (*n* = 2,157) were detected in routine diagnostics. A share of 15.8% (*n* = 340 in 335 patients) was identified as VREfm, with a considerable variation by month, ranging from 7.3% in March 2022 to 26.4% in June 2024.

PFGE analysis showed that 61.2% (*n* = 208) of primary isolates were part of the outbreak cluster peaking in the third quartile of 2023, while 33.2% (*n* = 113) were either independent or belonged to smaller clusters. Of 5.6% (*n* = 19), no isolate was available for further analysis or the isolates were non-typeable. The outbreak strain was classified as CC17/ST80 VREfm by WGS. Overall, the resistance to vancomycin was primarily induced by *vanA* (39/40 isolates analyzed by WGS). The remaining isolate acquired vancomycin resistance via *vanB*. This was underlined by the fact that 84.1% of isolates were resistant to teicoplanin (*n* = 286). It appeared that the combination of the genes *aph(3′)-IIIa*, *esp*, *hyl*, *tetL*, *tetM,* and *vanA,* and the absence of both *ecbA* and the plasmid rep14a (CP006625.1) was a pattern unique to the outbreak cluster.

### Rapid and reliable bacterial typing by MALDI-TOF MS

**Fig 2:**
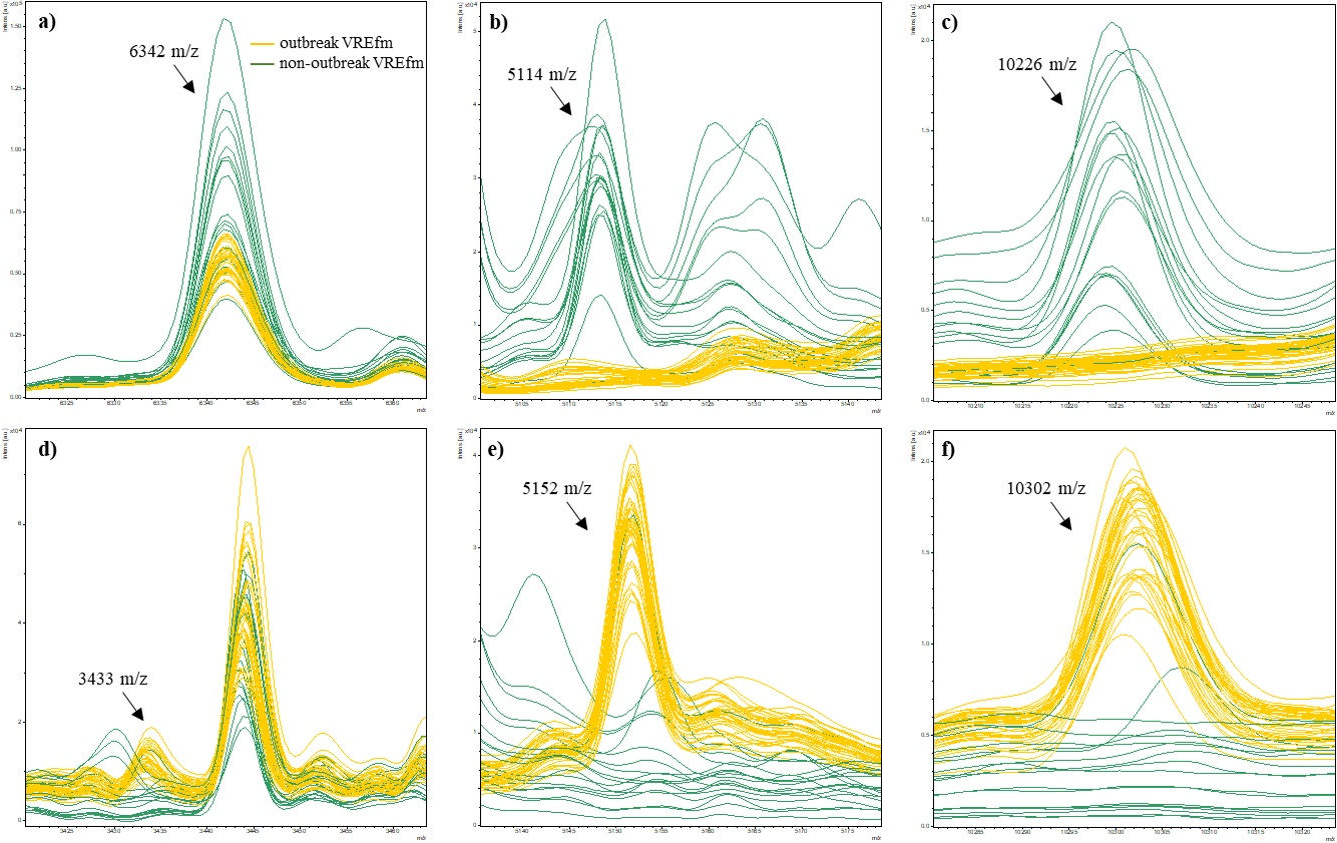
The specific peak pattern of the VREfm outbreak cluster strains was used to establish MALDI-TOF typing protocol, originally classified by PFGE (*n* = 60). Peaks include (**a**) the common reference peak at 6,342 *m/z* used for mass spectra alignment, absence of specific peaks at (**b**) 5,114 *m/z* and (**c**) 10,226 *m/z*, and presence of specific peaks for a/a* isolates at (**d**) 3,433 *m/z*, (**e**) 5,152 *m/z*, and (**f**) 10,302 *m/z*. Lines represent mass spectra for outbreak isolates (yellow) and non-outbreak isolates (green).

Based on the initial visual evaluation of the mass spectra (*n* = 40) as well as literature research (7, 18), a specific peak pattern for the outbreak strains was identified. It is characterized by the presence of peaks at 3,433, 5,152, and 10,302 *m/z* as well as the absence of peaks at 5,114 and 10,226 *m/z* (Fig. 2; Table S2).

Since peak 3,433 *m/z* was the least pronounced, it was excluded for further analysis. For the proof of concept and its robustness, isolates were independently measured three times, and a Python code was generated to automatically read the extractable mass list of technical triplicates. For classification as an outbreak (related) strain, four requirements had to be met: (i) A minimal signal-to-noise ratio (SNR) of 6 with a tolerance in the *m/z* of ±2 to assure signal quality, (ii) presence of the *E. faecium* control peak (6,342 *m/z*) for calibration of the spectra, (iii) presence of peaks at 5,152 and 10,302 *m/z*, and (iv) absence of peaks at 5,114 and 10,226 *m/z*, both for specificity to the predefined outbreak strain pattern. Subsequently, four possible read-outs were defined as follows: (i) strain probably related to outbreak strain, (ii) strain most likely unrelated to outbreak strain, (iii) further investigation required (PFGE), and (iv) result invalid (recalibration needed/no *E. faecium*).

The comparison of VREfm typing using MALDI-TOF MS against PFGE showed 59 concordant results and one false-positive result, resulting in a preliminary sensitivity of 1.00 (95% CI: 0.92–1.00), specificity of 0.94 (0.74–1.00), and accuracy of 0.98 (0.91–1.00) (Table 1).

**TABLE 1:** Primary evaluation of the newly established typing model^*a*^^,^^*b*^

*n* = 60	Predicted positive (%)	Predicted negative (%)	Test performance
True positive	42 (70.0%)	0 (0.0%)	100.0% Sensitivity
True negative	1 (1.7%)	17 (28.3%)	94.4% Specificity
	97.7% PPV	100.0% NPV	98.4% Accuracy

^*a*^ NPV, negative predictive value; PPV, positive predictive value.

^*b*^ Comparison of VREfm typing by PFGE results versus MALDI-TOF MS.

To determine the reproducibility of the method, three independent runs on different days of biological replicates were compared. Only one isolate (#8) displayed a discordant result (“further investigation required” in run 3) (Table S3). Hence, the method showed reproducible results as demonstrated by a mean accuracy of 97.83 ± 0.91% (mean ± SD) for the 60 initial isolates.

Most of the strains showed consistent results across the two different typing methods (PFGE as reference standard and MALDI-TOF MS). Only for isolate #24 a contradictory result was obtained; however, it showed a congruent result by MALDI-TOF MS and kSNP4 analyses (Table 2).

**TABLE 2:** Comparison of the three different typing methods for VREfm^*b*^

Isolate #	MLST	MLST (Bez)	PFGE	MALDI	kSNP4
7	1299	na	0^*a*^	0	0
12	1299	na	0^*a*^	0	0
13	80	1152	0	0	1
14	80	1152	1	1	1
15	80	1152	1	1	1
16	80	1152	0	0	1
17	80	na	1	1	1
18	80	1152	1	1	1
19	80	1152	1	1	1
20	80	1152	1	1	1
21	80	1152	1	1	1
22	80	1152	1	1	1
23	80	1152	1	1	1
24	80	1152	0	1	1
25	80	1152	1	1	1
26	80	1152	1	1	1
27	80	1152	1	1	1
28	80	152	0	0	0
29	80	1152	1	1	1
30	80	1152	1	1	1
31	80	1152	1	1	1
32	80	1152	1	1	1
33	80	1152	0	0	1
34	80	1152	1	1	1
35	80	1152	1	1	1
36	80	1152	1	1	1
37	80	1152	1	1	1
38	80	1152	1	1	1
39	80	1152	1	1	1
40	80	1152	1	1	1
41	80	1152	1	1	1
42	80	1152	1	1	1
43	80	1152	1	1	1
44	117	17	0	0	0
45	80	1152	1	1	1
46	80	1152	1	1	1
47	80	1152	1	1	1
48	80	1152	1	1	1
49	80	na	0	0	0
50	80	1152	1	1	1

^*a*^ Non-typable, but based on WGS result counted as non-outbreak isolate (0). na: not matching any known MLST (Bezdicek) variant.

^*b*^ Methods included pulsed-field gel electrophoresis (PFGE) as gold standard, MALDI-TOF, and SNP-based analysis in order to group isolates into outbreak (1) or non-outbreak (0).

In a final step, the method was validated with 278 VREfm isolates, including those used for the initial establishment and accuracy analysis, by comparing the MALDI-TOF results to the gold standard PFGE. Thereby, the novel typing method showed a sensitivity of 1.00 (0.98–1.00) and a specificity of 0.79 (0.71–0.85) (Table 3).

**TABLE 3:** Validation of the newly established typing model^*a*^^,^^*b*^

*n* = 278	Predicted positive (%)	Predicted negative (%)	Test performance
True positive	182 (65.5%)	0 (0.0%)	100.0% Sensitivity
True negative	26 (9.3%)	70 (25.2%)	78.7% Specificity
	87.5% PPV	100.0% NPV	91.4% Accuracy

^*a*^ NPV, negative predictive value; PPV, positive predictive value.

^*b*^ Comparison of VREfm typing by PFGE results versus MALDI-TOF MS.

### Successful optimization and implementation of the novel MALDI-TOF MS typing protocol

Based on these promising results, an optimization attempt (see “Optimization and real-life application of VREfm strain typing by MALDI-TOF MS,” above) was performed to shorten the protocol for routine implementation. Hereby, 72 isolates were tested using the complete extraction protocol compared to the optimized protocol. Decreasing the minimal SNR from 6 to 4 led to a correct classification of all isolates. Using Müller-Hinton II agar as an alternative cultivation medium to Columbia III blood agar produced invalid and false-negative results with both extraction approaches in a subgroup of samples (*n* = 20) (Table S5), and was thus not continued. Overall, applying the optimized on-target protein extraction with adapted criteria for the SNR, the test demonstrated a sensitivity, specificity, and accuracy of 1.00 (95% CI: 0.98–1.00), 0.89 (0.70–0.97), and 0.96 (0.90–0.99), respectively (Table 4). The optimized method demonstrated a short hands on time (HOT) of approximately 30 minutes. The time-to-result (TTR) of the complete typing approach arises from the successful subcultivation of positive culture of new VREfm isolates on Columbia III blood agar and is hence approximating 1 day.

**TABLE 4:** Performance of optimized typing model in a real-life setting^*a*^^,^^*b*^

*n* = 72	Predicted positive (%)	Predicted negative (%)	Test performance
True positive	44 (61.1%)	0 (0.0%)	100.0% Sensitivity
True negative	3 (4.2%)	25 (34.7%)	89.3% Specificity
	93.6% PPV	100.0% NPV	95.8% Accuracy

^*a*^ NPV, negative predictive value; PPV, positive predictive value.

^*b*^ Comparison of VREfm typing by PFGE results versus direct MALDI-TOF MS.

The method was implemented in our routine diagnostics laboratory in November 2024 to rapidly identify outbreak cluster isolates and to promote targeted infection control measures in the respective hospital wards. Since then, a total of 126 first VREfm isolates (from 123 patients) were analyzed using the optimized protocol. Among these, 73.02% (*n* = 92) were classified as cluster isolates, with one patient even carrying both a cluster and a non-cluster isolate.

## DISCUSSION

VREfm contributes significantly to healthcare-associated infections, with CC17 strains being particularly noteworthy for their frequent involvement in hospital outbreaks. Since 2022, an increased detection of VREfm isolates has been observed at the Institute of Hygiene and Medical Microbiology, Medical University of Innsbruck. Genotyping by PFGE revealed that most of these isolates (61.2%) belonged to one distinct cluster. Based on WGS data derived from a subset of these isolates, this outbreak cluster was identified as a specific CC17/ST80 *vanA* lineage. VREfm outbreaks, primarily driven by *vanA*-associated strains, have been globally reported, with significant clusters of ST80, ST117, and ST796 emerging in European countries (19–22).

To ensure the prompt application of adequate outbreak control measures, rapid and reliable genotyping of the isolates is required, mainly obtained by PFGE or WGS. Nevertheless, genotyping results are often available only with a considerable lag time, and both of these methods come with significant drawbacks of labor- and/or time-intensity and high cost (6). With our study, a successful protocol for rapid and reliable strain typing using MALDI-TOF MS was established for the emerging CC17/ST80 VREfm *vanA* cluster. Hereby, a distinct peak pattern was identified for the outbreak strain, characterized by the presence of peaks at 3,433, 5,152, and 10,302 *m/z*, along with the absence of peaks at 5,114 and 10,226 *m/z*. No previously described cluster-specific peaks were present in these strains (7, 18). The established method was successfully evaluated (*n* = 60) with a reproducible accuracy of 97.83 ± 0.91% (mean ± SD) and validated (*n* = 278) with a sensitivity of 1.00 and specificity of 0.79. Discrepant results, as for isolate #24, potentially occurred due to the presence of mixed cultures containing both outbreak and independent strains. This is supported by the fact that kSNP4 analysis classified this isolate as an outbreak strain, and the observation of at least five patients carrying two different strain types simultaneously or consecutively. It has previously been demonstrated that patients can be colonized with multiple genetically diverse VREfm strains or diverging lineages (23, 24). Using the optimized protocol with direct on-target extraction and adapted criteria for peak analysis showed a sensitivity and specificity of 1.00 and 0.89, respectively, when compared to PFGE. The method was established using a relatively small initial sample set. As the sample pool expands and becomes more heterogeneous, it is expected that specificity may decrease due to the inclusion of unknown or atypical isolates. The relatively low specificity may be explained by false-positive isolates sharing similar attributes as the outbreak cluster. Preliminary WGS analyses (*n* = 10) suggest that some, but not all of the false-positive isolates show a close relation (two to three alleles) to cluster isolates identified by PFGE and actually would be considered as cluster isolates by genomic typing (data not shown). Future studies using a larger sample size and detailed WGS analyzes shall give more insights into the accuracy of the MALDI-TOF MS typing method to identify outbreak isolates on an allele level.

With a short HOT of approximately 30 min and a TTR of 1 day after culture of new VREfm isolates, this protocol has significant advantages over traditional typing methods. While MALDI-TOF MS-based typing has been previously used successfully for identifying cluster isolates in *Pseudomonas aeruginosa* outbreaks (25, 26), in VREfm outbreak settings, it demonstrated contradictive results ranging from good performance regarding relatedness (27) to limited practical value due to low specificity (7), and an overall accuracy comparable to PFGE (8). The discrimination of outbreak isolates from non-related strains is thus not uniformly applicable, but rather has to be performed according to strain-specific characteristics. Other rapid VREfm strain typing methods include the detection of vancomycin resistance genes to identify *vanA*/*vanB* subtypes by PCR (28), and MALDI-TOF MS (29). One recent study demonstrated the successful implementation of a PCR-based identification of ST117-CT469 outbreak strains, after primary PCR targeting *vanA/vanB* operons (30). In our strains, a customized PCR-based approach would have included several marker genes, requiring the establishment of several distinct PCRs. While the utilization of MALDI-TOF MS spectra in conjunction with machine learning algorithms has been demonstrated to effectively predict the presence of vancomycin resistance. In *E. faecium* (29), the present study is, to the best of our knowledge, the first to demonstrate the successful classification of outbreak strains within the VREfm population using MALDI-TOF MS.

WGS is frequently used as a potent tool for outbreak investigations demonstrated in a recent outbreak of an emerging VREfm ST612 *vanA* lineage in Switzerland (31). WGS is proposed to be the most reliable, though most expensive, typing method investigating nosocomial transmissions (32); however, it requires interpretation and translation into hygiene measures.

The established MALDI-TOF MS typing protocol bears several limitations, including (i) the use of PFGE as an outdated reference method, (ii) the approach being matched to only this specific CC17/ST80 cluster and therefore potentially bearing mainly regional benefit. Further, (iii) it cannot discriminate non-outbreak isolates from each other, and (iv) being an endpoint method that does not include evolving strains, leading to (v) the necessity to regularly reevaluate the established peak patterns.

However, due to the ready availability of the data and sufficient discriminatory power of the PFGE method, the comparison was appraised to be feasible. The genotyping of a subset of the isolates further corroborated these findings. The limitations have also been outweighed (at least in part) by the promptness and reliability of the novel MALDI-TOF MS results. The pressing need for timely information about the presence of outbreak strains on a daily basis subsequently led to the implementation of this method in routine diagnostics for the duration of the ongoing CC17/ST80 VREfm *vanA* outbreak. A first conclusion of this implemented method showed a positivity rate of 73.0% (*n* = 92). Taking into account the specificity of 89.3% for the optimized protocol, the estimated number of true-positive isolates is 82, corresponding to 65.1% cluster isolates. This closely reflects the proportion observed prior to the implementation of MALDI-TOF MS typing (61.2%). Future outbreak investigations on VREfm or also other concerning microbes might profit from this approach by applying the here established protocol to a collection of isolates.

Finally, this study shows the successful development of this rapid MALDI-TOF MS-based typing approach, which enabled the real-life application in routine diagnostics in an outbreak setting. This provided timely information about the presence of CC17/ST80 *vanA* VREfm outbreak isolates, which facilitates accurate infection prevention and control measures. Future studies will determine the accuracy of this method at the allele level and include ST80 isolates from variable regions within Europe to assess the potential of this method on a larger scale.

## Data Availability

The supplemental material provides information supporting our results. Upon reasonable request, the complete data set of this study is available from the corresponding authors (S.F. and M.G.).

## SUPPLEMENTAL MATERIAL

Supplemental tables spectrum.02702-25-s0001.pdf Tables S1 to S4.

ASM does not own the copyrights to Supplemental Material that may be linked to, or accessed through, an article. The authors have granted ASM a non-exclusive, world-wide license to publish the Supplemental Material files. Please contact the corresponding author directly for reuse.
