# Supplementary material for: Novel, rapid, and reliable typing of vancomycin-resistant Enterococcus faecium CC17/ST80 strains using MALDI-TOF MS
Source: Microbiol Spectr. 2025 Oct 1;13(11):e02702-25. doi: 10.1128/spectrum.02702-25 (PMC12584701; doi:10.1128/spectrum.02702-25)
Supplement: Supplemental tables — Tables S1 to S4. [file spectrum.02702-25-s0001.pdf]

Supplementary Material

Novel, rapid, and reliable typing of a distinct vancomycin-resistant *Enterococcus faecium* CC17/ST80 strain using MALDI-TOF MS

Silke Huber[1], Christina Brühwasser[2], David Eisele[1], Cornelia Lass-Flörl[1], Stefan Fuchs[1,#], Miriam Govrins[1,#]

[1]Institute of Hygiene and Medical Microbiology, Medical University of Innsbruck, Innsbruck, Austria

[2]Infection Prevention and Hospital Hygiene, University Hospital Innsbruck, Innsbruck, Austria

Supplementary Table S1. Overview of investigated genes for antimicrobial resistance (n=34), virulence factors (n=19), and plasmids (n=15) including accession code from the National Center for Biotechnology Information (NCBI) or the European Nucleotide Archive (ENA) to find a pattern specific to the emerging VRE CC17/ST80. Genes with a query coverage of at least 90% and an identity of at least 90% were counted as present. For the plasmids, the query coverage threshold was lowered to 50% due to some contigs in the assembled genomes being shorter than the plasmid sequence lengths.

**AMR Genes**	**Accession (NCBI)**
aac6-Ie_aph(2')-Ia	MZ485322.1
aph(3')-IIIa	KF767841.1
cfr	OQ117760.1
ddl	AY489046.1
eatA	KF010778.1
ermA	MZ539566.1
ermB	MW436601.1
ermC	MW436602.1
ireK	NZ_JBCGWY010000489.1
mefA	MN548247.1
msrC	MW554493.1
optrA	MT122999.1
parC	AB017811.1
pbp5	KJ742835.1
poxtA	MK292894.1
rpoB	AY526617.1
tetK	MG669282.1
tetL	MW436605.1
tetM	MW436604.1
tetO	AY660532.1
tetS	JN980096.1
vanA	PP829270.1
vanB	AY697424.1
vanC	NG_048353.1
vanD	NG_048362.1
vanE	AF136925.1
vanG	NG_048369.1
vanL	NG_048395.1
vanM	NG_048396.1
vanN	NG_048397.1
vatD	MG669289.1
vatE	AY043213.1
vatH	NG_048551.1
vgaD	NG_048560.1

**Virulence Factors**	**Accession (NCBI)**
ace	MW436613.1
acm	NZ_CP038996.1:c2144684-2142519
agg	OQ984057.1
asa1	KY654068.1
ccf	KX198029.1
cob	KU311661.1
cpd	KU311666.1
cylA	KY654069.1
cylB	KU311664.1
ecbA	NC_021994.1:c1914381-1911154
efaA	KY070337.1
efaAfm	FJ609170.1
esp	MW436612.1
fss3	NZ_CP038996.1:2660366-2663593
gelE	MW436611.1
gyrA	AF060881.1
hyl	KY654070.1
scm	NZ_CP038996.1:2754144-2756126
sgrA	NZ_CP038996.1:c1452606-1451632

**Plasmids**	**Accession (ENA)**
rep1	U83488.1
rep11a	AB178871.1
rep14a	CP003354.1
rep14a	EFU01917.1
rep14a	CP006625.1
rep14b	EU327398.1
rep17	AF507977.1
rep18a	AB158402.1
rep18b	AF408195.1
rep18b	CP018068.1
rep2	X92945.2
rep22	X03408.1
repUS12	AF181950.1
repUS15	CP004064.1
repUS43	CP003584.1

Supplementary Table S2. Investigated Peaks to find a discriminatory peak pattern for the distinct CC17-ST80 vancomycin-resistant *Enterococcus faecium* (VREfm) cluster (outbreak). Pulsed-field gel electrophoresis (PFGE) classified strains into cluster outbreak (1) or independent (0). Mass spectra (n=40) were evaluated visually and informed by literature [24, 25], leading to the investigation of 47 peaks within the 3000–12000 m/z range for their presence (1) or absence (0). A unique peak pattern for the outbreak cluster was identified, characterized by the presence of peaks at 3433, 5152, and 10302 m/z and the absence of peaks at 5114 and 10226 m/z. Isolates were further classified into cluster outbreak (1) or independent (0) based on MALDI-TOF MS analysis.

#	WGS	PFGE	MALDI	3253	3297	3300	3314	3341	3370	3373	3433	3444	3516	3644	3877	3924	4164	4428	4777	4812	4828	4887	5056	5062	5114	5152	5155	5196	5355	5701	5760	5784	5973	5990	5992	6051	6342	6354	6359	6509	6596	6602	6630	6684	6829	6890	7031	7289	7753	7848	8327	8981	9060	9552	9771	10226	10302	10308	10494	10941
7	1299	**0**	0	0	0	0	1	0	0	0	**0**	1	1	1	0	0	1	1	1	0	0	1	0	1	**1**	**0**	0	1	1	0	0	1	1	1	0	1	1	0	1	0	0	0	1	0	1	1	1	1	0	0	1	1	1	1	1	**1**	**0**	0	1	1
8	n.a.	**0**	0	1	0	0	1	0	0	0	**0**	1	1	1	0	0	1	1	1	0	0	1	0	1	**1**	**0**	0	1	1	0	0	1	1	1	0	1	1	0	1	1	0	0	1	0	1	1	1	1	0	0	1	1	1	1	1	**1**	**0**	0	1	1
12	1299	**0**	0	0	0	0	1	0	0	1	**0**	1	1	1	0	0	1	1	1	0	0	1	0	1	**1**	**0**	0	1	1	0	0	1	1	2	0	1	1	0	1	0	0	0	1	0	1	1	1	1	0	0	1	1	1	1	1	**1**	**0**	0	1	1
13	80	**0**	0	0	1	1	1	0	1	0	**0**	1	0	1	0	0	1	1	1	1	1	0	1	0	**1**	**0**	1	1	1	0	0	1	1	0	1	0	1	0	1	0	1	0	1	0	1	1	0	1	0	1	1	1	1	1	0	**1**	**0**	1	1	1
16	80	**0**	0	1	0	0	1	0	0	1	**0**	1	1	1	0	0	1	1	1	0	0	1	1	0	**1**	**0**	0	1	1	0	0	1	1	1	0	1	1	0	1	1	0	0	1	0	1	1	1	1	0	0	1	1	1	1	1	**1**	**0**	0	1	1
24	80	**0**	1	1	0	0	1	0	0	1	**1**	1	1	1	0	0	1	1	1	0	0	1	1	0	**0**	**1**	0	1	1	0	0	1	1	1	0	1	1	0	1	1	0	0	1	0	1	1	1	1	0	1	1	1	1	1	1	**0**	**1**	0	1	1
28	80	**0**	0	1	0	0	0	0	0	1	**0**	1	1	1	1	0	1	1	1	0	0	1	1	0	**1**	**0**	0	1	1	0	1	0	1	1	0	1	1	0	1	1	0	1	0	0	1	1	1	1	1	2	1	1	1	1	1	**1**	**0**	0	1	1
33	80	**0**	0	1	0	0	1	0	0	1	**0**	1	1	1	0	0	1	1	1	0	0	1	0	1	**1**	**0**	0	1	1	0	0	1	1	1	0	1	1		1	1	0	0	1	0	1	1	1	1	0	0	1	1	1	1	1	**1**	**0**	0	1	1
44	117	**0**	0	1	0	1	0	0	0	0	**0**	1	1	1	2	0	1	1	1	0	0	1	0	1	**1**	**0**	0	1	1	2	0	1	1	1	0	1	1	0	1	1	0	1	0	0	1	1	1	1	1	0	1	1	1	1	1	**1**	**0**	0	1	1
49	80	**0**	0	1	0	1	0	0	1	0	**0**	1	1	1	0	0	1	1	1	0	0	1	0	0	**1**	**0**	0	1	1	2	0	1	1	1	0	0	1	0	1	1	0	1	0	0	1	1	1	1	0	2	1	2	2	2	2	**1**	**0**	0	2	2
51	n.a.	**0**	0	1	0	1	0	1	0	0	**0**	1	1	1	0	0	1	1	1	0	0	1	0	1	**1**	**0**	0	1	1	0	0	1	1	1	0	1	1	0	1	1	0	1	0	0	1	1	1	1	0	0	1	1	1	1	1	**1**	**0**	0	2	2
52	n.a.	**0**	0	1	0	1	0	1	0	0	**0**	1	1	1	0	0	1	1	1	0	0	1	0	0	**1**	**0**	0	1	2	0	0	2	1	1	0	1	1	0	1	1	0	1	0	0	2	1	1	1	0	0	1	2	1	1	1	**1**	**0**	0	2	2
53	n.a.	**0**	0	1	0	0	1	0	0	2	**0**	1	1	1	0	0	1	1	1	0	0	1	0	1	**1**	**0**	0	1	1	0	0	1	1	1	0	1	1	0	1	1	0	0	1	0	1	1	1	1	0	0	1	1	1	1	1	**1**	**0**	0	1	2
54	n.a.	**0**	0	1	0	1	0	1	0	2	**0**	1	1	1	0	0	1	1	1	0	0	1	0	0	**1**	**0**	0	1	1	0	0	1	1	1	0	1	1	0	1	1	0	1	0	0	1	1	1	1	0	0	1	1	1	1	1	**1**	**0**	0	2	2
55	n.a.	**0**	0	1	0	1	0	0	0	2	**0**	1	1	1	0	0	1	1	1	0	0	1	0	1	**1**	**0**	0	1	1	0	0	1	1	1	0	1	1	0	1	1	0	1	0	0	1	1	1	1	0	0	1	1	1	1	1	**1**	**0**	0	2	2
56	n.a.	**0**	0	1	0	1	0	1	0	2	**0**	1	1	1	0	0	1	1	1	0	0	1	0	2	**1**	**0**	0	1	1	0	0	1	1	1	0	1	1	0	1	1	0	1	0	0	1	1	1	1	0	0	1	1	1	1	1	**1**	**0**	0	2	2
57	n.a.	**0**	0	1	0	0	1	0	0	0	**0**	1	1	1	0	0	1	1	1	0	0	1	0	0	**1**	**0**	0	1	1	1	0	1	1	1	0	1	1	0	1	1	0	0	1	0	1	1	1	1	0	0	1	1	1	1	1	**1**	**0**	0	2	2
58	n.a.	**0**	0	1	0	1	0	1	0	2	**0**	1	1	1	0	0	1	1	1	0	0	1	0	1	**1**	**0**	0	1	1	0	0	1	1	1	0	1	1	0	1	1	0	1	0	0	1	1	1	1	0	0	1	1	1	1	1	**1**	**0**	0	2	2
1	n.a.	**1**	1	1	0	0	1	0	0	1	**1**	1	1	1	0	0	1	1	1	0	0	1	0	1	**0**	**1**	0	1	1	0	0	1	1	1	0	1	1	0	1	1	0	0	1	0	1	1	1	1	0	0	1	1	1	1	1	**0**	**1**	0	1	1
2	n.a.	**1**	1	1	0	0	1	0	0	1	**1**	1	1	1	0	0	1	1	1	0	0	1	0	1	**0**	**1**	0	1	1	0	0	1	1	1	0	1	1	0	1	1	0	0	1	0	1	1	1	1	0	0	1	1	1	1	1	**0**	**1**	0	1	1
3	n.a.	**1**	1	1	0	0	1	0	0	1	**1**	1	1	1	0	0	1	1	1	0	0	1	0	1	**0**	**1**	0	1	1	0	0	1	1	1	0	1	1	0	1	1	0	0	1	0	1	1	1	1	0	0	1	1	1	1	1	**0**	**1**	0	1	1
4	n.a.	**1**	1	1	0	0	1	0	0	1	**1**	1	1	1	0	0	1	1	1	0	0	1	0	1	**0**	**1**	0	1	1	0	0	1	1	1	0	1	1	0	1	1	0	0	1	0	1	1	1	1	0	0	1	1	1	1	1	**0**	**1**	0	1	1
5	n.a.	**1**	1	1	0	0	1	0	0	1	**1**	1	1	1	0	0	1	1	1	0	0	1	0	1	**0**	**1**	0	1	1	0	0	1	1	1	0	1	1	0	1	1	0	0	1	0	1	1	1	1	0	0	1	1	1	1	1	**0**	**1**	0	1	1
6	n.a.	**1**	1	1	0	0	1	0	0	1	**1**	1	1	1	0	0	1	1	1	0	0	1	0	1	**0**	**1**	0	1	1	0	0	1	1	1	0	1	1	0	1	1	0	0	1	0	1	1	1	1	0	0	1	1	1	1	1	**0**	**1**	0	1	1
9	n.a.	**1**	1	1	0	0	1	0	0	1	**1**	1	1	1	0	0	1	1	1	0	0	1	0	1	**0**	**1**	0	1	1	0	0	1	1	1	0	1	1	0	1	1	0	0	1	0	1	1	1	1	0	0	1	1	1	1	1	**0**	**1**	0	1	1
10	n.a.	**1**	1	1	0	0	1	0	0	1	**1**	1	1	1	0	0	1	1	1	0	0	1	0	1	**0**	**1**	0	1	1	0	0	1	1	1	0	1	1	0	1	1	0	0	1	0	1	1	1	1	0	0	1	1	1	1	1	**0**	**1**	0	1	1
11	n.a.	**1**	1	1	0	0	1	0	0	1	**1**	1	1	1	0	0	1	1	1	0	0	1	0	1	**0**	**1**	0	1	1	0	0	1	1	1	0	1	1	0	1	1	0	0	1	0	1	1	1	1	0	0	1	1	1	1	1	**0**	**1**	0	1	1
14	80	**1**	1	1	0	0	1	0	0	1	**1**	1	1	1	0	0	1	1	1	0	0	1	1	0	**0**	**1**	0	1	1	0	0	1	1	1	0	1	1	0	1	1	0	0	1	0	1	1	1	1	0	0	1	1	1	1	1	**0**	**1**	0	1	1
15	80	**1**	1	1	0	0	1	0	0	1	**1**	1	1	1	0	0	1	1	1	0	0	1	1	0	**0**	**1**	0	1	1	0	0	1	1	1	0	1	1	0	1	1	0	0	1	0	1	1	1	1	0	1	1	1	1	1	1	**0**	**1**	0	1	1
17	80	**1**	1	1	0	0	1	0	0	1	**1**	1	1	1	0	0	1	1	1	0	0	1	1	0	**0**	**1**	0	1	1	0	0	1	1	1	0	1	1	0	1	1	0	0	1	0	1	1	1	1	0	0	1	1	1	1	1	**0**	**1**	0	1	1
18	80	**1**	1	1	0	0	1	0	0	1	**1**	1	1	1	0	0	1	1	1	0	0	1	1	0	**0**	**1**	0	1	1	0	0	1	1	1	0	1	1	0	1	1	0	0	1	0	1	1	1	1	0	0	1	1	1	1	1	**0**	**1**	0	1	1
19	80	**1**	1	1	0	0	1	0	0	1	**1**	1	1	1	0	0	1	1	1	0	0	1	1	0	**0**	**1**	0	1	1	0	0	1	1	1	0	1	1	0	1	1	0	0	1	0	1	1	1	1	0	0	1	1	1	1	1	**0**	**1**	0	1	1
20	80	**1**	1	1	0	0	1	0	0	1	**1**	1	1	1	0	0	1	1	1	0	0	1	1	0	**0**	**1**	0	1	1	0	0	1	1	1	0	1	1	0	1	1	0	0	1	0	1	1	1	1	0	0	1	1	1	1	1	**0**	**1**	0	1	1
21	80	**1**	1	1	0	0	1	0	0	1	**1**	1	1	1	0	0	1	1	1	0	0	1	1	0	**0**	**1**	0	1	1	0	0	1	1	1	0	1	1	0	1	1	0	0	1	0	1	1	1	1	0	0	1	1	1	1	1	**0**	**1**	0	1	1
22	80	**1**	1	1	0	0	1	0	0	1	**1**	1	1	1	0	0	1	1	1	0	0	1	1	0	**0**	**1**	0	1	1	0	0	1	1	1	0	1	1	0	1	1	0	0	1	0	1	1	1	1	0	0	1	1	1	1	1	**0**	**1**	0	1	1
23	80	**1**	1	1	0	0	1	0	0	1	**1**	1	1	1	0	0	1	1	1	0	0	1	1	0	**0**	**1**	0	1	1	0	0	1	1	1	0	1	1	0	1	1	0	0	1	0	1	1	1	1	0	0	1	1	1	1	1	**0**	**1**	0	1	1
25	80	**1**	1	1	0	0	1	0	0	1	**1**	1	1	1	0	0	1	1	1	0	0	1	1	0	**0**	**1**	0	1	1	0	0	1	1	1	0	1	1	0	1	1	0	0	1	0	1	1	1	1	0	0	1	1	1	1	1	**0**	**1**	0	1	1
26	80	**1**	1	1	0	0	1	0	0	1	**1**	1	1	1	0	0	1	1	1	0	0	1	1	0	**0**	**1**	0	1	1	0	0	1	1	1	0	1	1	0	1	1	0	0	1	0	1	1	1	1	0	0	1	1	1	1	1	**0**	**1**	0	1	1
27	80	**1**	1	1	0	0	1	0	0	1	**1**	1	1	1	0	0	1	1	1	0	0	1	1	0	**0**	**1**	0	1	1	0	0	1	1	1	0	1	1	0	1	1	0	0	1	0	1	1	1	1	0	0	1	1	1	1	1	**0**	**1**	0	1	1
29	80	**1**	1	1	0	0	1	0	0	1	**1**	1	1	1	0	0	1	1	1	0	0	1	1	0	**0**	**1**	0	1	1	0	0	1	1	1	0	1	1	0	1	1	0	0	1	0	1	1	1	1	0	0	1	1	1	1	1	**0**	**1**	0	1	1
30	80	**1**	1	1	0	0	1	0	0	1	**1**	1	1	1	0	0	1	1	1	0	0	1	1	0	**0**	**1**	0	1	1	0	0	1	1	1	0	1	1	0	1	1	0	0	1	0	1	1	1	1	0	0	1	1	1	1	1	**0**	**1**	0	1	1
31	80	**1**	1	1	0	0	1	0	0	1	**1**	1	1	1	0	0	1	1	1	0	0	1	1	0	**0**	**1**	0	1	1	0	0	1	1	1	0	1	1	0	1	1	0	0	1	0	1	1	1	1	0	0	1	1	1	1	1	**0**	**1**	0	1	1
32	80	**1**	1	1	0	0	1	0	0	1	**1**	1	1	1	0	0	1	1	1	0	0	1	1	0	**0**	**1**	0	1	1	0	0	1	1	1	0	1	1	0	1	1	0	0	1	0	1	1	1	1	0	0	1	1	1	1	1	**0**	**1**	0	1	1
34	80	**1**	1	1	0	0	1	0	0	1	**1**	1	1	1	0	0	1	1	1	0	0	1	0	1	**0**	**1**	0	1	1	0	0	1	1	1	0	1	1	0	1	1	0	0	1	0	1	1	1	1	0	2	1	1	1	1	1	**0**	**1**	0	1	1
35	80	**1**	1	1	0	0	1	0	0	1	**1**	1	1	1	0	0	1	1	1	0	0	1	0	1	**0**	**1**	0	1	1	0	0	1	1	1	0	1	1	0	1	1	0	0	1	0	1	1	1	1	0	0	1	1	1	1	1	**0**	**1**	0	1	1
36	80	**1**	1	1	0	0	1	0	0	1	**1**	1	1	1	0	0	1	1	1	0	0	1	0	1	**0**	**1**	0	1	1	0	0	1	1	1	0	1	1	0	1	1	0	0	1	0	1	1	1	1	0	0	1	1	1	1	1	**0**	**1**	0	1	1
37	80	**1**	1	1	0	0	1	0	0	1	**1**	1	1	1	0	0	1	1	1	0	0	1	0	1	**0**	**1**	0	1	1	0	0	1	1	1	0	1	1	0	1	1	0	0	1	0	1	1	1	1	0	0	1	1	1	1	1	**0**	**1**	0	1	1
38	80	**1**	1	1	0	0	1	0	0	1	**1**	1	1	1	0	0	1	1	1	0	0	1	0	1	**0**	**1**	0	1	1	0	0	1	1	1	0	1	1	0	1	1	0	0	1	0	1	1	1	1	0	0	1	1	1	1	1	**0**	**1**	0	1	1
39	80	**1**	1	1	0	0	1	0	0	1	**1**	1	1	1	0	0	1	1	1	0	0	1	0	1	**0**	**1**	0	1	1	0	0	1	1	1	0	1	1	0	1	1	0	0	1	0	1	1	1	1	0	0	1	1	1	1	1	**0**	**1**	0	1	1
40	80	**1**	1	1	0	0	1	0	0	1	**1**	1	1	1	0	0	1	1	1	0	0	1	0	1	**0**	**1**	0	1	1	0	0	1	1	1	0	1	1	0	1	1	0	0	1	0	1	1	1	1	0	0	1	1	1	1	1	**0**	**1**	0	1	1
41	80	**1**	1	1	0	0	1	0	0	1	**1**	1	1	1	0	0	1	1	1	0	0	1	0	1	**0**	**1**	0	1	1	0	0	1	1	1	0	1	1	0	1	1	0	0	1	0	1	1	1	1	0	0	1	1	1	1	1	**0**	**1**	0	1	1
42	80	**1**	1	1	0	0	1	0	0	1	**1**	1	1	1	0	0	1	1	1	0	0	1	0	1	**0**	**1**	0	1	1	0	0	1	1	1	0	1	1	0	1	1	0	0	1	0	1	1	1	1	0	0	1	1	1	1	1	**0**	**1**	0	1	1
43	80	**1**	1	1	0	0	1	0	0	1	**1**	1	1	1	0	0	1	1	1	0	0	1	0	1	**0**	**1**	0	1	1	0	0	1	1	1	0	1	1	0	1	1	0	0	1	0	1	1	1	1	0	0	1	1	1	1	1	**0**	**1**	0	1	1
45	80	**1**	1	1	0	0	1	0	0	1	**1**	1	1	1	0	0	1	1	1	0	0	1	0	1	**0**	**1**	0	1	1	0	0	1	1	1	0	1	1	0	1	1	0	0	1	0	1	1	1	1	0	0	1	1	1	1	1	**0**	**1**	0	1	1
46	80	**1**	1	1	0	0	1	0	0	1	**1**	1	1	1	0	0	1	1	1	0	0	1	0	1	**0**	**1**	0	1	1	0	0	0	1	1	0	1	1	0	1	1	0	0	1	0	1	1	1	1	0	0	1	1	1	1	1	**0**	**1**	0	1	1
47	80	**1**	1	1	0	0	1	0	0	1	**1**	1	1	1	0	0	1	1	1	0	0	1	0	1	**0**	**1**	0	1	1	0	0	1	1	1	0	1	1	0	1	1	0	0	1	0	1	1	1	1	0	0	1	1	1	1	1	**0**	**1**	0	1	1
48	80	**1**	1	1	0	0	1	0	0	1	**1**	1	1	1	0	0	1	1	1	0	0	1	0	1	**0**	**1**	0	1	1	0	0	1	1	1	0	1	1	0	1	1	0	0	1	0	1	1	1	1	0	0	1	1	1	1	1	**0**	**1**	0	1	1
50	80	**1**	1	1	0	0	1	0	0	1	**1**	1	1	1	0	0	1	1	1	0	0	1	0	1	**0**	**1**	0	2	1	0	0	1	1	1	0	1	1	0	1	1	0	0	1	0	1	1	1	1	0	0	1	1	1	1	1	**0**	**1**	0	1	2
59	n.a.	**1**	1	1	0	0	1	0	0	2	**1**	1	1	1	0	0	1	1	1	0	0	1	0	1	**0**	**1**	0	1	1	0	0	1	1	1	0	1	1	0	1	1	0	0	1	0	1	1	1	1	0	0	1	1	1	1	1	**0**	**1**	0	1	2
60	n.a.	**1**	1	1	0	0	1	0	0	2	**1**	1	1	1	0	0	1	1	1	0	0	1	0	1	**0**	**1**	0	1	1	0	0	1	1	1	0	1	1	0	1	1	0	0	1	0	1	1	1	1	0	0	1	1	1	1	1	**0**	**1**	0	1	2

#: isolate number, WGS: sequence type (ST) from whole genome sequence data, n.a. not analyzed

Supplementary Table S3. Reproducability of VRE typing by MALDI-TOF MS. Isolates were classified in outbreak (1) or non-outbreak (0) based on three independent analyses. One isolate gained in one out of the three runs the result “further investigation” (2).

**Isolate #**	**PFGE**	**MALDI**	**MALDI (automatic analysis)**		
			**Run 1**	**Run 2**	**Run 3**
1	1	1	1	1	1
2	1	1	1	1	1
3	1	1	1	1	1
4	1	1	1	1	1
5	1	1	1	1	1
6	1	1	1	1	1
7	n/t	0	0	1	0
**8**	**0**	**0**	**0**	**0**	**2**
9	1	1	1	1	1
10	1	1	1	1	1
11	1	1	1	1	1
12	n/t	0	0	0	0
13	0	0	0	0	0
14	1	1	1	1	1
15	1	1	1	1	1
16	0	0	0	0	0
17	1	1	1	1	1
18	1	1	1	1	1
19	1	1	1	1	1
20	1	1	1	1	1
21	1	1	1	1	1
22	1	1	1	1	1
23	1	1	1	1	1
24	0	1	1	1	1
25	1	1	1	1	1
26	1	1	1	1	1
27	1	1	1	1	1
28	0	0	0	0	0
29	1	1	1	1	1
30	1	1	1	1	1
31	1	1	1	1	1
32	1	1	1	1	1
33	0	0	0	0	0
34	1	1	1	1	1
35	1	1	1	1	1
36	1	1	1	1	1
37	1	1	1	1	1
38	1	1	1	1	1
39	1	1	1	1	1
40	1	1	1	1	1
41	1	1	1	1	1
42	1	1	1	1	1
43	1	1	1	1	1
44	0	0	0	0	0
45	1	1	1	1	1
46	1	1	1	1	1
47	1	1	1	1	1
48	1	1	1	1	1
49	0	0	0	0	0
50	1	1	1	1	1
51	0	0	0	0	0
52	0	0	0	0	0
53	0	0	0	0	0
54	0	0	0	0	0
55	0	0	0	0	0
56	0	0	0	0	0
57	0	0	0	0	0
58	0	0	0	0	0
59	1	1	1	1	1
60	1	1	1	1	1

n/t: not typable, however classified as ST1299 by WGS and thereby non-a/a

Supplementary Table S4. An alternative agar plate (Müller-Hinton; MH) for bacterial cultivation was tested for their effect on the MALDI-TOF MS results, using the standard protein extraction and the optimized protocol with direct formic acid treatment (n=20).

**Isolate #**	**PFGE**	**MALDI**	**MALDI MH**	**MALDI MH direct**
168	1	1	1	1
290	1	1	1	1
302	1	1	1	1
**303**	**1**	**1**	**1**	**0**
**304**	**0**	**0**	**x**	**x**
305	0	0	0	0
306	1	1	1	1
307	1	1	1	1
308	1	1	1	1
309	1	1	1	1
310	0	1	1	1
311	0	1	1	1
288	0	0	0	0
289	1	1	1	1
291	1	1	1	1
292	1	1	1	1
293	0	0	0	0
**294**	**0**	**0**	**x**	**0**
297	0	0	0	0
**299**	**0**	**0**	**x**	**0**

1: outbreak strain; 0: non-outbreak, x: invalid result (control peak 6342 m/z not found)
